# Adverse Events and Perception of Benefit From Duloxetine for Treating Aromatase Inhibitor-Associated Arthralgias

**DOI:** 10.1093/jncics/pkab018

**Published:** 2021-02-08

**Authors:** Patrick M Schnell, Maryam B Lustberg, N Lynn Henry

**Affiliations:** 1 Division of Biostatistics, The Ohio State University College of Public Health, Columbus, OH, USA; 2 Division of Internal Medicine, The Ohio State University College of Medicine, Columbus, OH, USA; 3 Department of Internal Medicine, Division of Hematology/Oncology, University of Michigan Medical School, Ann Arbor, MI, USA

## Abstract

**Background:**

Duloxetine effectively treats aromatase inhibitor-associated musculoskeletal symptoms (AIMSS) in women with breast cancer but causes low-grade toxicities. This secondary analysis examines the relationship between adverse events (AE) and patient-perceived benefit, based on patient self-report that the treatment received was beneficial despite side effects. We hypothesized that duloxetine had a favorable effect on patient-perceived benefit, even among duloxetine-treated patients who experienced AEs and who, had they been treated with placebo, would have experienced none.

**Methods:**

Principal stratification was used to estimate the effect of duloxetine vs placebo on patient-perceived benefit and Functional Assessment of Cancer Therapy-Endocrine Scale functional quality of life in the randomized, double-blind trial SWOG S1202 (n = 289). Subgroups of patients were defined by observed and counterfactual (what would have occurred had they been randomly assigned to the opposite study arm) experiences of AEs and the original primary outcome, reduction of average pain after 12 weeks of at least 2 points on the Brief Pain Inventory-Short Form.

**Results:**

Duloxetine caused an estimated 23.4% (95% credible interval [CI] = 13.4% to 33.7%) of patients to experience an AE even though they would have experienced none on placebo. Those patients remained more likely to report that their received treatment was beneficial than comparable patients assigned placebo (73.3% vs 41.8%, respectively; 95% CI for difference = 15.4 to 47.2 percentage points), although there was no statistically significant effect of duloxetine on functional quality of life (11.3 vs 9.0, 95% CI for difference = -2.2 to +6.7).

**Conclusion:**

Duloxetine resulted in higher patient-perceived benefit, even among those who would have an AE on duloxetine but none on placebo. Treatment of AIMSS with duloxetine should be considered for appropriate patients.

Aromatase inhibitors (AIs) are an effective treatment for hormone receptor–positive early-stage breast cancer (1). However, up to half of treated patients develop aggravating AI-associated musculoskeletal symptoms (AIMSS) such as joint pain and stiffness (2). Intolerance to AI therapy causes 20% to 30% of patients to discontinue treatment early, and of those who discontinue treatment, 75% do so because of AIMSS (2).

Duloxetine is a serotonin-norepinephrine reuptake inhibitor used for the treatment of depression and anxiety, as well as several chronic pain conditions, including chronic musculoskeletal pain, but is not Food and Drug Administration–approved for AIMSS (3). The SWOG S1202 randomized, placebo-controlled trial of duloxetine for the treatment of AIMSS in postmenopausal women with early-stage breast cancer demonstrated that duloxetine reduced average joint pain compared with placebo within 12 weeks (4).

Despite efficacy of duloxetine in treating AIMSS, widespread use may be limited by the association of duloxetine with an increased rate of adverse events (AEs). In S1202, patients randomly assigned to duloxetine had higher rates of AEs of any grade compared with placebo (78% vs 50%) (4). However, more patients in the duloxetine arm compared with the placebo arm reported that their received treatment was beneficial with either no or limited AEs or despite AEs (71% vs 49%) (4). Although these results indicate that duloxetine was considered more beneficial overall, the relationship between pain reduction, AEs, and patient-perceived benefit is unclear.

If it is assumed that duloxetine causes but does not prevent AEs, many patients would have had AEs regardless of treatment assignment (50%, the proportion experiencing AEs in the placebo arm), potentially because of the specific population studied or the context of the trial, or would have had no AEs regardless of treatment assignment (22%, the proportion not experiencing AEs in the duloxetine arm). Only the difference in AE rates between treatment arms would be attributed to duloxetine (28%). It is meaningful to know whether the difference in rates of patient-perceived benefit was solely because of pain reduction when treatment assignment did not influence whether they experienced AEs, or if—even among patients for whom assignment to duloxetine caused them to have an AE when they otherwise would not have—the reductions in pain outweighed the AEs to the extent that they resulted in a higher rate of patient-perceived benefit from treatment.

Previous reports from the S1202 trial do not fully describe the relationship between AEs and patient-reported outcomes such as reductions in pain, patient perception of benefit, and functional quality of life (FQOL). It is not known whether those who experienced AEs were more likely to report positive outcome or whether duloxetine caused positive outcomes in those patients who would experience an AE on duloxetine but not on placebo. We hypothesized that AEs would be positively correlated with pain reduction within each treatment group and that duloxetine would have positive effects on patient perception of benefit and FQOL even among patients who would experience an AE on duloxetine but not on placebo.

## Methods

### Data Acquisition

After obtaining a letter of exemption from the Ohio State University institutional review board, data were obtained from the National Clinical Trials Network [NCTN]–National Cancer Institute Community Oncology Research Program (NCORP) Data Archive of the National Cancer Institute’s (NCI’s) NCTN. Data were originally collected from the SWOG S1202 clinical trial NCT01598298. A description of the trial, including complete eligibility criteria and study design, has been published previously (4).

### Eligibility

The S1202 trial was approved by the institutional review boards of the participating institutions. Postmenopausal women who had been receiving AI therapy for at least 3 weeks and no more than 24 months, and with average joint pain of at least 4 out of 10 on the Brief Pain Inventory (BPI) (5) that either developed while taking AI therapy or worsened since AI initiation, were eligible. Those patients whose pain was due to fracture or traumatic injury were ineligible. All subjects provided written informed consent (4).

### Study Design

Patients were randomly assigned 1:1 to receive duloxetine or placebo. Patients assigned to duloxetine received 30 mg daily for 1 week followed by 60 mg daily for 11 weeks. Patients assigned to placebo received matching sugar capsules. Patients and physicians were blinded to treatment allocation. Patients completed questionnaires at baseline and at 2, 6, 12, and 24 weeks.

### Outcome Measures

Patients were classified as having an AE if they reported an AE of any grade as defined by the S1202 protocol (4) other than arthralgia. Arthralgias were excluded because of close association to the eligibility criteria and the anticipated therapeutic effect of duloxetine. Patients were classified as experiencing a clinically significant reduction in pain if their average pain score assessed by the BPI (0-10 scale) decreased by at least 2 points from baseline to week 12, as was used for the primary endpoint of the trial (6).

At week 12, patients were asked to classify the received blinded treatment as beneficial with limited or no side effects, beneficial despite side effects, not worth the side effects, or not yielding improvement in symptoms. We define patient perception of benefit as “beneficial” if the patient stated that the treatment was beneficial with limited or no side effects or beneficial despite side effects. Participants who declared “none of the above” or “prefer not to answer” were classified as not reporting a perceived benefit. Patients were also asked whether they believed they had been receiving duloxetine or placebo.

FQOL was assessed via the Functional Assessment of Cancer Therapy-Endocrine Scale (FACT-ES) (7). Change in FACT-ES functional well-being subscale score from baseline to week 12 was used as the FQOL outcome.

### Participant Characteristics

**Figure 1.**
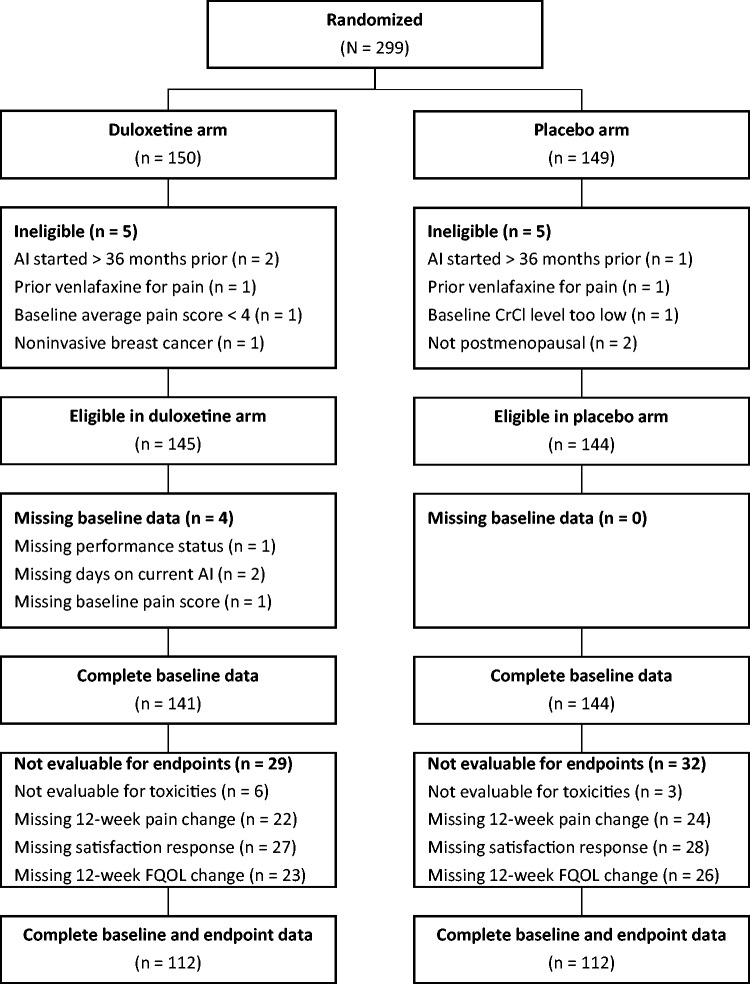
CONSORT diagram. AI = aromatase inhibitor; CrCI = creatinine clearance; FQOL = functional quality of life.

Of the 299 participants randomly assigned in S1202, 289 were eligible for the trial, and of those, 141 in the duloxetine group and 144 in the placebo group had complete baseline data (see Figure 1). Baseline characteristics among participants with complete baseline data by treatment group are provided in Table 1. All factors were well balanced between treatment groups. Baseline characteristics for all participants randomly assigned and eligible for S1202 have been reported previously (4). Of those patients, 112 in the duloxetine group and 112 in the placebo group had complete outcome data. The 285 participants with complete baseline data were used in all subsequent analyses except for the tabulation of adverse events, for which the 279 eligible participants evaluable for toxicities (138 on duloxetine, 141 on placebo) were used regardless of the completeness of baseline data.

**Table 1.** Baseline characteristics of participants included in the primary analysis dataset (eligible and with complete baseline data)^a^

Characteristic	Duloxetine (n = 141)	Placebo (n = 144)	Overall (N = 285)
Age, y			
Median (min, max)	60.0 (40.0, 83.0)	60.0 (27.0, 82.0)	61.0 (27.0, 83.0)
Hispanic, No. (%)			
Yes	5 (3.5)	6 (4.2)	11 (3.9)
No	136 (96.5)	138 (95.8)	274 (96.1)
Race, No. (%)			
White	124 (87.9)	120 (83.3)	244 (85.6)
Black	10 (7.1)	17 (11.8)	27 (9.5)
Other	7 (5.0)	7 (4.9)	14 (4.9)
ECOG performance status, No. (%)			
0	99 (70.2)	94 (65.3)	193 (67.7)
1	41 (29.1)	48 (33.3)	89 (31.2)
2	1 (0.7)	2 (1.4)	3 (1.1)
Nodal involvement, No. (%)			
Negative	97 (68.8)	89 (61.8)	186 (65.3)
Positive	44 (31.2)	55 (38.2)	99 (34.7)
Baseline pain score, No. (%)			
46	106 (75.2)	110 (76.4)	216 (75.8)
710	35 (24.8)	34 (23.6)	69 (24.2)
Prior taxane use, No. (%)			
No	65 (46.1)	65 (45.1)	130 (45.6)
Yes	76 (53.9)	79 (54.9)	155 (54.4)
Duration of current AI therapy, days			
Median (min, max)	265 (7, 1050)	258 (21, 1100)	261 (7, 1100)
Total AI medications, No. (%)			
1	116 (82.3)	119 (82.6)	235 (82.5)
2	21 (14.9)	23 (16.0)	44 (15.4)
3	4 (2.8)	2 (1.4)	6 (2.1)
Prior tamoxifen use, No. (%)			
No	123 (87.2)	124 (86.1)	247 (86.7)
Yes	18 (12.8)	20 (13.9)	38 (13.3)

a AI = aromatase inhibitor; ECOG = Eastern Cooperative Oncology Group.

### Statistical Analysis

All analyses were performed using R (version 4.0.2). The primary analysis was restricted to patients with all included baseline variables measured (n = 285). Missing values of AE occurrence (n = 9, 3.2%) and pain reduction (n = 46, 16.1%) were imputed using Bayesian multiple imputation with a multinomial logistic regression model with vague priors (Gaussian with mean [SD] = 0 [10]) to allow the use of available baseline and other outcome data under the missing-at-random assumption. Credible intervals (CI) were constructed at the 95% level.

The primary prespecified aim was to determine the effect of duloxetine on patient perception of benefit within strata defined by observed and counterfactual outcomes in adverse events and reductions in pain. Because the counterfactual outcomes are not observed, patients cannot be classified into strata based on observed data alone; thus, traditional methods for stratified data, such as the Cochran-Mantel-Haenszel test, cannot be used. We define basic principal strata (8) as subgroups of patients based on whether they would have experienced AEs on either duloxetine or placebo (always), duloxetine only (duloxetine), or neither duloxetine nor placebo (never), as well as whether they would have experienced a reduction in pain of at least 2 points on the BPI on either duloxetine or placebo (always), duloxetine only (duloxetine), or neither duloxetine nor placebo (never). Because each patient’s experiences are observed on only one of duloxetine or placebo, the patient’s counterfactual (unobserved because of assignment to the opposite arm) experiences are considered missing data and handled via Bayesian multiple imputation. Sensitivity analyses are performed to evaluate the consequences of stratification by baseline pain category and prior taxane use, as well as violation of assumptions employed by the principal stratification analysis.

We adopt a commonly used assumption of monotonicity: if a patient would experience an AE on placebo, then they would also experience an AE on duloxetine, and similarly for reductions in pain. This assumption allows us to estimate the likelihood that each patient belongs to principal strata using the principal scores technique (9): we train a classification model based on observed pain reductions and AEs in each arm using multinomial logistic regression and use the trained classifier to perform Bayesian multiple imputation of the experiences patients would have had if they had been allocated to the opposite trial arm. As predictors of AEs and reductions in pain, we use age at baseline, ethnicity, race, Eastern Cooperative Oncology Group performance status, nodal involvement, average pain at baseline, prior taxane use, time on current AI therapy, total number of different AI medications taken, and prior tamoxifen use. We estimate the proportion of the population within each principal stratum, as well as treatment effects on patient perception of benefit, belief that they had been receiving duloxetine, and FQOL within those strata. In addition to reporting comparison-wise credible intervals, we produce credible intervals adjusted for simultaneous estimation in the 9 principal strata for each endpoint (10) and report whether they include zero. We used a beta prior with both shape parameters set to 0.5 for binary outcome distributions (assignment guess and perceived benefit) and a Gaussian-inverse-gamma prior with center parameter zero and shape and rate parameters 0.01 for the means and variances of the FQOL distributions within principal strata. We fit the model using 100 000 Gibbs sampler iterations after 1000 burn-in iterations. Plots of posterior means of *t* statistics comparing covariate means between treatment arms within each stratum were used to confirm successful balancing of covariates by the principal score model.

The secondary prespecified aim was to determine the associations of AEs and positive patient-reported outcomes within each treatment group. We stratify by treatment group and compare positive patient-reported outcomes between those who reported AEs and those who did not using *t* tests and χ^2^ tests for marginal comparisons and linear and logistic regression for baseline-adjusted comparisons, as appropriate for the type of outcome. All frequentist tests were 2-sided, and a *P* value of less than .05 was considered statistically significant. All credible intervals were 2-sided, and differences for which the 95% credible intervals did not contain the point of no difference (eg, a difference of 0 or odds ratio of 1) were considered statistically significant.

## Results

### Adverse Events

Of the 279 eligible patients evaluable for AEs, 108 (78.3%) in the duloxetine arm and 68 (48.2%) in the placebo arm experienced at least 1 AE other than arthralgia. Table 2 displays the occurrences per arm of AE types of any grade experienced by at least 10% of patients in either arm. The most common AEs in the duloxetine arm were fatigue (31.9% vs 12.8% on placebo), nausea (30.4% vs 6.4% on placebo), and dry mouth (25.4% vs 12.8% on placebo). Of the 10 AE types shown, each was experienced by a larger proportion of patients in the duloxetine arm than in the placebo arm. Grade 3 AEs recorded were fatigue, insomnia, hypersomnia, pain, pain in extremity, myalgia, nausea, vomiting, diarrhea, headache, and decreased range of motion. Grade 3 AEs other than arthralgia were experienced by 11 patients (8.0%) in the duloxetine arm and 4 patients (2.8%) in the placebo arm.

**Table 2.** Adverse events of all grades

Adverse event	Duloxetine, No. (%) (n = 138)	Placebo, No. (%) (n = 141)
Any (other than arthralgia)	108 (78.3)	68 (48.2)
Occurred in >10% of patients in either group		
Fatigue	44 (31,9)	18 (12.8)
Nausea	42 (30.4)	9 (6.4)
Dry mouth	35 (25.4)	18 (12.8)
Headache	29 (21.0)	18 (12.8)
Myalgia	21 (15.2)	10 (7.1)
Hot flashes	20 (14.5)	12 (8.5)
Insomnia	19 (13.8)	7 (5.0)
Diarrhea	18 (13.0)	6 (4.3)
Dizziness	18 (13.0)	4 (2.8)
Constipation	17 (12.3)	7 (5.0)

### Effects of Duloxetine in Principal Strata

Table 3 displays estimates and 95% credible intervals for observed rates of patient-perceived benefit and changes in FQOL by assignment group. The proportion of participants reporting a perceived benefit was higher in the duloxetine group than the placebo group (71.8% vs 49.1%, 95% CI for difference = 9.6 to 35.7 percentage points). There was no statistically significant difference in change in FQOL between groups (12.3 vs 11.5, 95% CI for difference = -2.4 to 4.0).

**Table 3.** Observed outcomes at 12 weeks^a^

Endpoint	Duloxetine	Placebo	Difference, pp (95% CI)
Patient perception of benefit (%)	84 (71.8)	57 (49.1)	23 (9.6 to 35.7)
Change in functional quality of life (FACT-ES TOI)	12.3	11.5	0.8 (-2.4 to 4.0)

a FACT-ES TOI = Functional Assessment of Cancer Therapy-Endocrine Scale trial outcome index; pp = percentage points.

**Figure 2.**
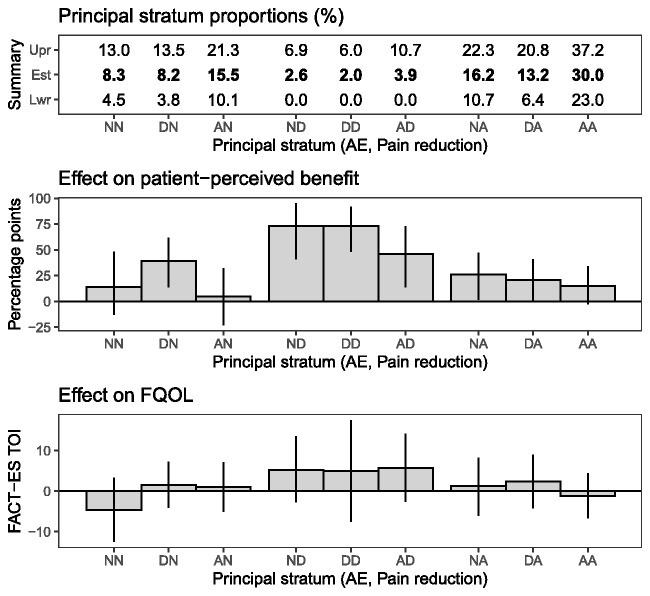
Posterior means (Estimate [“Est”] or bar height) and 95% credible intervals (Lower/Upper [“Lwr”/”Upr”] or vertical lines) for population proportion (**top**) and effects of duloxetine on patient-perceived benefit (**middle**) and functional QOL (**bottom**) by AE and pain reduction principal strata. Strata are defined by whether patients would have experienced an AE in neither arm (never), only on duloxetine (duloxetine), or in either arm (always), and similarly for pain reduction. For example, the ND stratum consists of patients who would not have an AE in either arm (never) and would experience pain reduction only on duloxetine (duloxetine). Positive differences in patient-perceived benefit and FQOL indicate favorable effects of duloxetine. Credible intervals for the effect on patient-perceived benefit for strata NA and DA included 0 when adjusted for multiple comparisons, but pointwise credible intervals did not. AA = always-always; AD = always-duloxetine; AE = adverse event; AN = always-never; DA = duloxetine-always; DD = duloxetine-duloxetine; DN = duloxetine-never; FQOL = functional quality of life trial outcome index; NA = never-always; ND = never-duloxetine; NN = never-never.

Figure 2 displays estimates and 95% credible intervals for relative sizes of principal strata in the study population and the effect of duloxetine on patient perception of benefit and FQOL. Strata are defined by whether patients would have experienced an AE in neither arm (Never), only on duloxetine (Duloxetine), or in either arm (Always), and similarly for pain reduction. Strata are denoted by the AE stratum first and pain reduction stratum second and abbreviated by the first letter of each stratum. For example, the ND stratum consists of patients who would not have an AE in either arm (Never) and would experience pain reduction only on duloxetine (Duloxetine). Most patients’ AE and pain reduction statuses would not be influenced by treatment assignment: strata NN, NA, AN, and AA together (70.1%, 95% CI = 57.0% to 82.6%). A large percentage of patients would experience both AEs and pain reduction on either duloxetine or placebo: stratum AA (30.0%, 95% CI = 23.0% to 37.2%). The estimated proportion of the population who would experience at least 1 AE on duloxetine but no AEs on placebo (strata DN, DD, and DA together) (23.4%, 95% CI = 13.4% to 33.7%) is larger than for those who would experience a reduction in pain on duloxetine but not placebo (strata ND, DD, and AD together) (8.5%, 95% CI = 0.3% to 20.4%). However, the evidence for a difference is inconclusive (95% CI = -29.1 to 0.6 percentage points [pp]).

**Figure 3.**
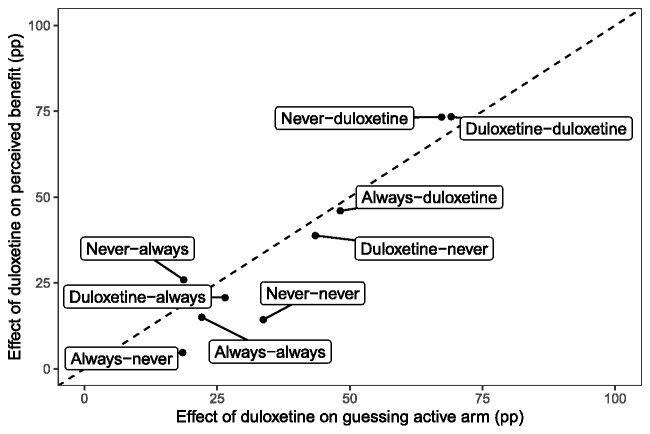
Effects of duloxetine on patients guessing that they were in the active treatment arm and on patient-perceived benefit. Posterior mean effects (percentage point [pp] difference) of duloxetine on patients guessing that they were in the active treatment arm and on patient-perceived benefit. Principal strata are labeled by adverse events first and pain reduction second (eg, patients in the “never-duloxetine” stratum would not have an adverse event regardless of arm assignment and would have a reduction in pain only if assigned to the duloxetine arm). Above the diagonal, the effect on perceived benefit is stronger than on patients’ guesses, and below the diagonal, it is weaker.

When group assignment affects neither AE status nor pain reduction status (strata NN, NA, AN, and AA), there is little evidence of a treatment effect on patient perception of benefit, except among patients who would never have an AE but always a reduction in pain (stratum NA), for whom there was a 25.9 pp difference (95% CI = 1.7 to 47.3 pp) in perceived benefit. When assignment to duloxetine causes a reduction in pain, it has a positive effect on patient perception of benefit regardless of whether the patient would have no adverse events on either duloxetine or placebo (stratum ND) (73.3 pp, 95% CI = 41.2 to 95.4 pp), an AE on duloxetine but not placebo (stratum DD) (73.4 pp, 95% CI = 47.7 to 92.7 pp), or an AE on either (stratum AD) (46.0 pp, 95% CI = 13.9 to 73.3 pp). Among patients for whom duloxetine but not placebo causes an AE, assignment to duloxetine has a positive but smaller effect on patient perception of benefit even when patients would have had a reduction in pain in either arm (stratum DA) (20.7 pp, 95% CI = 0.8 to 40.9 pp) or in neither arm (stratum DN) (38.8 pp, 95% CI = 14.2 to 61.5 pp). Credible intervals adjusted for multiple comparisons excluded 0 for all statistically significant results described above except for those in strata NA and DN. Table 4 displays treatment effects in principal strata defined by AE statuses only. Among all patients who would have an AE under duloxetine but not placebo, the effect of duloxetine on patient-perceived benefit was favorable (31.5 pp, 95% CI = 15.4 to 47.2 pp). The effects of duloxetine in principal strata on patient belief that they had been receiving duloxetine (vs placebo) mirror the effects on perception of benefit but are generally more extreme (Figure 3). Stratified and sensitivity analyses yielded results consistent with the primary analyses (Supplementary Methods, Supplementary Data File, available online).

**Table 4.** Marginal adverse event (AE) principal stratum treatment effects^a^

Endpoint	AE stratum	Duloxetine	Placebo	Difference (95% CI for difference)
Patient perception of benefit (%)	Never	68.2	41.3	26.9 (7.7 to 44.8)^b^
	Duloxetine	73.3	41.8	31.5 (15.4 to 47.2)^b^
	Always	72.0	57.7	14.3 (-0.5 to 29.0)
Change in functional quality of life (FACT-ES TOI)	Never	10.2	10.3	-0.1 (-5.4 to 5.0)
	Duloxetine	11.3	9.0	2.3 (-2.2 to 6.8)
	Always	12.3	12.3	0.0 (-4.0 to 4.0)

a Principal stratum treatment effects reported as per-arm posterior means and comparison-wise 95% credible intervals (CIs). Treatment effect on perceived benefit (percentage point difference), and treatment effect on functional quality of life (FACT-ES TOI score difference). FACT-ES TOI = Functional Assessment of Cancer Therapy-Endocrine Scale trial outcome index.

b Credible intervals adjusted for multiplicity of principal strata do not contain zero.

There are no statistically significant effects on FQOL in strata defined by the combination of AE and pain reduction statuses alone (Figure 2) or in strata defined by AE status alone (Table 4). In particular, among patients who would have had an AE under duloxetine but not placebo, the effect of duloxetine on FQOL was estimated to be 2.2 (FACT-ES trial outcome index; 95% CI = -2.2 to +6.8).

### Associations Between Adverse Events and Positive Outcomes

Within duloxetine and placebo groups separately, the occurrence of an AE was not associated with any patient perception of benefit, belief of randomization to duloxetine, reduction in pain, or FQOL, with or without adjustment for baseline variables. Table 5 displays estimates and credible intervals without adjustment. Differences in proportions of patients reporting a perceived benefit and a belief of randomization to duloxetine were positive but statistically insignificant. Credible intervals for differences in means of pain reduction and FQOL between patients who experienced AEs and those who did not suggest that any differences are clinically insignificant or nearly so. An ad hoc analysis of perceived benefit and specific AEs within each arm yielded no statistically significant associations after multiplicity correction (Supplementary Table 1, available online).

**Table 5.** Adverse event (AE) associations within study arms^a^

Outcome measure (AE vs no AE)	Placebo	Duloxetine
Reduction in pain difference in means (95% CI)	0.0 (-0.2 to 0.2)	0.0 (-0.2 to 0.2)
FQOL difference in means (95% CI)	2.5 (-1.9 to 6.9)	1.4 (-4.0 to 6.9)
Perception of benefit percentage point difference (95% CI)	11.4 (-5.9 to 28.7)	12.7 (-8.1 to 33.6)
Belief of randomization to duloxetine percentage point difference (95% CI)	13.9 (-5.9 to 33.6)	9.1 (-12.8 to 31.0)

a Estimates not adjusted for baseline variables. CI = credible interval; FQOL = functional quality of life.

## Discussion

A large proportion of patients assigned to placebo reported that their received treatment was beneficial. The AEs experienced in either arm were generally low grade, and a placebo effect may have resulted in a reduction in mean pain over time and therefore perceived benefit in both arms. Thus, a comparison of rates of patient-perceived benefit between arms is critical. Furthermore, many patients would have had an AE regardless of treatment assignment or would have had no AEs regardless of treatment assignment. Thus, a complete evaluation of the benefit-risk trade-off requires comparisons of rates of patient-perceived benefit among those who would have AEs on duloxetine but none on placebo.

The proportion of patients reporting that the reductions in pain experienced outweighed side effects (if any) was higher in the duloxetine arm than in the placebo arm. This difference was not driven solely by patients for whom duloxetine caused a reduction in pain but no AEs, as a favorable treatment effect was present even among patients who would have had AEs on duloxetine but none on placebo. Notably, a larger proportion of patients in the duloxetine arm than in the placebo arm reported that their received treatment was beneficial, even among patients for whom duloxetine would cause an AE but not a clinically significant reduction in pain. This result may be because of a type of placebo effect: patients who experience no AE and no pain reduction on control do not perceive a benefit, whereas those who experience an AE under duloxetine believe they are receiving the active treatment and expect and eventually report a benefit. This hypothesis is supported by the close relationship between treatment effects on perception of benefit and belief of randomization to duloxetine, although the direction of causation is not identifiable from these data.

These new analyses of the SWOG S1202 trial data provide evidence of a more universal benefit of duloxetine for the treatment of AIMSS with respect to patient-perceived benefit. Even among patients who would experience AEs on duloxetine but not on placebo, the benefit–-risk trade-off appears favorable to duloxetine. However, additional barriers to this use of duloxetine remain, including potential interactions with other drugs (3) and stigma associated with using a drug known to be an antidepressant (11). Duloxetine is one tool among several [eg, exercise (12) and acupuncture (13)] for management of AIMSS, and it will be important for treatment decision making to better understand which groups of patients benefit from which therapies. Finally, the relationship between perceived benefit and patients’ guesses about their random assignments indicates that collecting such data may be useful in interpreting data and guiding treatment recommendations based on symptom management trials, especially when there is considerable placebo response.

The results of the analyses presented here are subject to a few important limitations. First, only 75% of the patients eligible for the trial and randomly assigned to a study arm had complete data for the present analyses, although there do not appear to be any systematic differences between the patterns of missing data between arms. The multiple imputation approach to analyses with missing data partially mitigate this limitation. Second, a previously conducted exploratory analysis suggested that body mass index is associated with response to duloxetine or placebo, but these data were not available from the NCORP Data Archive. Finally, the principal scores approach to stratification used for the primary analysis relies on assumptions of monotonicity and general principal ignorability, the plausibility of which must be evaluated within each specific scientific context in which they are used. We have described the monotonicity assumption in an earlier section. The general principal ignorability assumption requires that, given the available baseline variables and observed adverse event and pain reduction statuses under treatment, the outcomes under treatment are independent of the counterfactual AE and pain reduction statuses under control, and vice versa. This assumption is comparable to assumptions of no unobserved confounders in analyses of observational data using propensity scores. The assumptions necessary for these analyses of data from a parallel arm study may be obviated by a crossover study in which intermediate and final outcomes may be observed under both treatment and control. However, such a study design would introduce other complications, particularly carryover effects.

## Funding

Patrick M Schnell was partially supported by the National Institutes of Health/National Center for Advancing Translational Sciences grant UL1TR002733.

## Notes

**Role of the funder:** The funding source did not participate in the design of the study; the collection, analysis, and interpretation of the data; the writing of the manuscript; or the decision to submit the manuscript for publication.

**Disclosures:** Dr Schnell has been a consultant to Merck & Co. Dr Lustberg has been a consultant to Disarm Therapeutics and Pledpharma. Dr Henry has received research support, paid to her institution, from Abbvie, Innocrin Pharmaceuticals, and Pfizer. Per NCTN/NCORP Data Archive policy, the submitted manuscript has been reviewed by the NCI and the sponsor of the original study, Eli Lilly & Company.

**Author contributions:** All authors of this research paper have directly participated in the planning, execution, and/or analysis of this study. Patrick M Schnell: conceptualization, formal analysis, methodology, software, visualization, and writing—original draft and review & editing. Maryam B Lustberg: conceptualization and writing—review & editing. N Lynn Henry: conceptualization and writing—review & editing.

**Acknowledgements:** This manuscript was prepared using data from Datasets NCT01598298-D1, NCT01598298-D2, and NCT01598298-D3 from the NCTN/NCORP Data Archive of the National Cancer Institute’s National Clinical Trials Network (NCTN). Data were originally collected from clinical trial NCT number NCT01598298, “A Randomized Placebo-Controlled Phase III Study of Duloxetine for Treatment of Aromatase Inhibitor-Associated Musculoskeletal Symptoms in Women With Early Stage Breast Cancer: SWOG S1202.”

Patrick M Schnell was partially supported by the National Institutes of Health/National Center for Advancing Translational Sciences grant UL1TR002733.

**Disclaimer:** All analyses and conclusions in this manuscript are the sole responsibility of the authors and do not necessarily reflect the opinions or views of the clinical trial investigators, the NCTN, the NCI, or the authors’ institutions.

## Data Availability

This manuscript was prepared using data from Datasets NCT01598298-D1, NCT01598298-D2, and NCT01598298-D3 from the NCTN/NCORP Data Archive of the National Cancer Institute’s (NCI’s) National Clinical Trials Network (NCTN). Data were originally collected from clinical trial NCT number NCT01598298, “A Randomized Placebo-Controlled Phase III Study of Duloxetine for Treatment of Aromatase Inhibitor-Associated Musculoskeletal Symptoms in Women With Early Stage Breast Cancer: SWOG S1202.” All analyses and conclusions in this manuscript are the sole responsibility of the authors and do not necessarily reflect the opinions or views of the clinical trial investigators, the NCTN, or the NCI.

## Supplementary Material

pkab018_Supplementary_Data
