# Supplementary material for: Adverse Events and Perception of Benefit From Duloxetine for Treating Aromatase Inhibitor-Associated Arthralgias
Source: JNCI Cancer Spectr. 2021 Feb 8;5(2):pkab018. doi: 10.1093/jncics/pkab018 (PMC8023424; doi:10.1093/jncics/pkab018)
Supplement: pkab018_Supplementary_Data [file pkab018_supplementary_data.zip › CS20-0135R1 Schnell supp mat_020321.pdf]

SUPPLEMENTARY MATERIAL

Adverse Events and Perception of Benefit from Duloxetine for Treating Aromatase Inhibitor–Associated Arthralgias

Patrick M Schnell*, PhD. The Ohio State University College of Public Health, Division of Biostatistics.

Maryam B Lustberg, MD, MPH. The Ohio State University College of Medicine, Division of Internal Medicine.

N Lynn Henry, MD, PhD. University of Michigan Medical School, Department of Internal Medicine, Division of Hematology/Oncology.

* Corresponding author: Patrick M Schnell (schnell.31@osu.edu; 1-614-292-5274)

SUPPLEMENTARY METHODS

Associations between adverse events and patient perception of benefit

Within each arm (duloxetine and placebo), Fisher's exact test was used to compare the proportions of patients with and without each adverse event who reported a perceived benefit. Because the occurrence of an adverse event is a post-randomization variable, patients who experience adverse events on placebo and on duloxetine are not directly comparable. The Bonferroni-corrected threshold for statistical significance is 0.0025.

Stratified analyses of the effects of duloxetine

Results of analyses stratified by baseline pain score category and prior taxane exposure, by which randomization was stratified in SWOG S1202 [2]. Stratification is applied to each variable separately to maintain reasonable sample sizes.

Estimates and lower and upper 95% credible interval bounds are presented. An asterisk (*) in the Sig. Adj. column indicates that the credible interval does not contain zero after adjustment for multiplicity of principal strata. [3]

Sensitivity analyses for violation of monotonicity

Results of sensitivity analyses for violation of the monotonicity assumption [1] are presented here. These sensitivity analyses are based on a parameter eta_ae, which is the ratio of the sizes of the stratum for which (for adverse events) placebo causes an adverse event when there wouldn't have been one on duloxetine, and the stratum for which duloxetine causes an adverse event when there wouldn't have been one on placebo (the former stratum assumed to be empty under the monotonicity assumption). A similar parameter, eta_pain, can be specified for the violation of monotonicity of pain reduction, and the conditional independence of pain reduction and adverse event intermediate outcomes can be applied straightforwardly.

Estimates and lower and upper 95% credible interval bounds are presented. An asterisk (*) in the Sig. Adj. column indicates that the credible interval does not contain zero after adjustment for multiplicity of principal strata. [3] In addition to the already-named strata, a (P)lacebo stratum is defined for patients who would have an adverse event (or pain reduction) on placebo but not on duloxetine.

Sensitivity analyses for violation of general principal ignorability (GPI)

Results of sensitivity analyses for violation of the general principal ignorability (GPI) assumption [1] are presented here. Under the monotonicity assumption, sensitivity analyses for GPI with respect to adverse events are implemented via parameters epsilon_1, describing the ratio of the expected outcome in the "duloxetine" stratum versus the "always" stratum under duloxetine, and epsilon_0, describing the ratio of the expected outcome in the "duloxetine" stratum versus the "never" stratum under placebo. Values of 1 correspond to the GPI assumption. Sensitivity analyses are presented for adverse events and pain reduction separately.

Estimates and lower and upper 95% credible interval bounds are presented. An asterisk (*) in the Sig. Adj. column indicates that the credible interval does not contain zero after adjustment for multiplicity of principal strata. [3]

Compilation of supplementary data

Results (estimates and 95% credible interval bounds) presented in the main text were reproduced under the stratified and sensitivity analyses, and tabulated in the **Supplementary Data File** (available online). The following data columns are presented:

1. `Observable_Stratum`: either the entire dataset ("All") or one of the prior taxane use or baseline pain strata used in the stratified analyses;
2. `Eta_AE`: Monotonicity sensitivity analysis parameter for adverse events;
3. `Eta_Pain`: Monotonicity sensitivity analysis parameter for pain reduction;
4. `GPI_Violation`: Which, if any, of the intermediate outcomes (AE, pain) violate the GPI assumption in the GPI sensitivity analysis;
5. `Epsilon_0`: GPI sensitivity analysis parameter for the placebo arm;
6. `Epsilon_1`: GPI sensitivity analysis parameter for the duloxetine arm;
7. `Parameter`: The parameter being estimated ("Population proportion", "Perceived benefit", or "Functional QOL")
8. `Group`: whether the parameter is estimated in one of the groups ("Duloxetine" or "Placebo"), the full sample ("Combined"), or as the absolute "Difference" between arms.
9. `Principal_Stratum`: As named in the main text (e.g., "DN" for patients who would have an adverse event on duloxetine but not placebo and would not have a reduction in pain on either), with an asterisk (*) in the second position indicating aggregation to AE principal strata only;
10. `Estimate`: posterior mean
11. `Lower_Bound_2_5`: 2.5% posterior quantile (lower bound of 95% CI);
12. `Upper_Bound_97_5`: 97.5% posterior quantile (upper bound of 95% CI).

References

[1] Ding, P and J. Lu, "Principal stratification analysis using principal scores," *Journal of the Royal Statistical Society: Series B (Statistical Methodology)*, vol. 79, no. 3, pp. 757-777, 2017.

[2] N. L. Henry, J. M. Unger, A. F. Schott, L. Fehrenbacher, P. J. Flynn, D. M. Prow, C. W. Sharer, G. V. Burton, C. S. Kuzma, A. Moseley, D. L. Lew, M. J. Fisch, C. M. Moinpour, D. L. Hershman and J. L. Wade, "Randomized, multicenter, placebo-controlled clinical trial of duloxetine versus placebo for aromatase inhibitor–associated arthralgias in early-stage breast cancer: SWOG S1202," *Journal of Clinical Oncology*, vol. 36, no. 4, pp. 326-332, 2018.

[3] P. M. Schnell, Q. Tang, W. W. Offen and B. P. Carlin, "A Bayesian credible subgroups approach to identifying patient subgroups with positive treatment effects," *Biometrics*, vol. 72, no. 4, pp. 1026-1036, 2016.

Supplementary Table 1. Associations between adverse events and patient perception of benefit

Adverse event	Study arm	% with AE benefiting	% without AE benefiting	Unadjusted p-value
Fatigue	Placebo	50	49	1.00
	Duloxetine	67	73	0.69
Nausea	Placebo	50	49	1.00
	Duloxetine	71	73	0.82
Dry mouth	Placebo	54	49	0.78
	Duloxetine	77	70	0.64
Headache	Placebo	60	48	0.42
	Duloxetine	68	73	0.79
Myalgia	Placebo	67	48	0.32
	Duloxetine	71	72	1.00
Hot flashes	Placebo	64	48	0.36
	Duloxetine	50	77	0.03
Insomnia	Placebo	83	47	0.11
	Duloxetine	53	75	0.08
Diarrhea	Placebo	40	50	1.00
	Duloxetine	67	73	0.76
Dizziness	Placebo	67	49	0.62
	Duloxetine	71	72	1.00
Constipation	Placebo	60	49	0.68
	Duloxetine	63	73	0.38

[a] Within each arm (duloxetine and placebo), Fisher's exact test was used to compare the proportions of patients with and without each adverse event who reported a perceived benefit. Because the occurrence of an adverse event is a post-randomization variable, patients who experience adverse events on placebo and on duloxetine are not directly comparable. The Bonferroni-corrected threshold for statistical significance is 0.0025.
